# Surgeon-modified fenestrated endovascular grafts and thoracoscope-assisted fixation for treatment of thoraco-abdominal aortic aneurysms

**DOI:** 10.1186/s13019-024-02686-y

**Published:** 2024-04-10

**Authors:** Hong Wang, Wei Wang, Weifan Wang, Debin Liu

**Affiliations:** 1 Department of Cardiac Surgery and Respiratory, Lanzhou University Second Hospital, Lanzhou, China; 2 Department of Cardiac Surgery, Hainan General Hospital, Hainan Hospital Affiliated to Hainan Medical University, No. 19, Xiuhua Road, Xiuying District, Haikou, Hainan China

**Keywords:** Surgeon-modified fenestrated stent graft, Thoracoabdominal aortic aneurysm, Thoracoscope-assisted fixation, Migration

## Abstract

**Background:**

Total endovascular technique with fenestrated endovascular graft might be hampered for the late dilatation of proximal landing zone, which may cause endografts migration. We describe a successful urgent hybrid procedure for extent III thoracoabdominal aortic aneurysm with aortic intramural hematoma.

**Case Presentation:**

A 55-year-old female with thoracoabdominal aortic aneurysm was considered at high surgical risk and unfit for open repair due to multiple comorbidities. Therefore, a hybrid procedure of surgeon-modified fenestrated endovascular graft combined with thoracoscope-assisted Transaortic epicardial fixation of endograft was finally chosen and performed in the endovascular operating room. A 3-port technique was performed through a left video-assisted thoracoscopic approach. After the first tampering stent-graft was deployed, a double-needle suture was penetrated both the aortic wall and stent-graft to fixate it in the proximal descending aorta. Then the second endograft, which had been fenestrated on table, was introduced and oriented extracorporeally by rotating superior mesenteric artery and left renal artery fenestration radiopaque markers and deployed with perfect apposition between the fenestrations and target visceral artery. Each vessel was sequentially stented using Viabahn self-expandable stent to finish target vessel stenting. An Ankura cuff stent was deployed in the distal abdominal aortic artery.

**Conclusion:**

Surgeon-modified fenestrated endovascular graft combined with thoracoscope-assisted fixation may be an innovative and viable alternative for selected high-risk patients with extent III thoracoabdominal aortic aneurysm. A longer follow-up is needed to ascertain the success of this approach.

## Background

Open repair of thoracoabdominal aortic aneurysm (TAAA) disease remains one of the most technically demanding operations. Despite progress in operative techniques, anatomically challenging aneurysms involving visceral arteries are still associated with substantial morbidity and mortality [1]. For this reason, fenestrated endovascular aortic repair (FEVAR) has become a widely used alternative for the treatment of TAAA in patients at high risk for open repair [2].

In thoracoabdominal endovascular aortic repair (TEVAR) there is a need for an acceptable length of “normal” or “healthy” aorta in the proximal and distal landing zone to have adequate fixation and seal of the endograft. Hybrid repair can be defined as a combined surgical and endovascular approach providing a suitable landing zone and simultaneous or staged TEVAR of the aneurysm. Most hybrid strategies only involve extra-anatomic bypass of the visceral vessels (“debranching”), however, video assisted thoracoscopy is rarely mentioned. This report describes a hybrid procedure which consists of an innovative thoracoscope-assisted fixation approach and surgeon-modified fenestrated endovascular graft (SMFEG) for electively high-risk patient with extent III TAAA.

## Case presentation

**Fig. 1:**
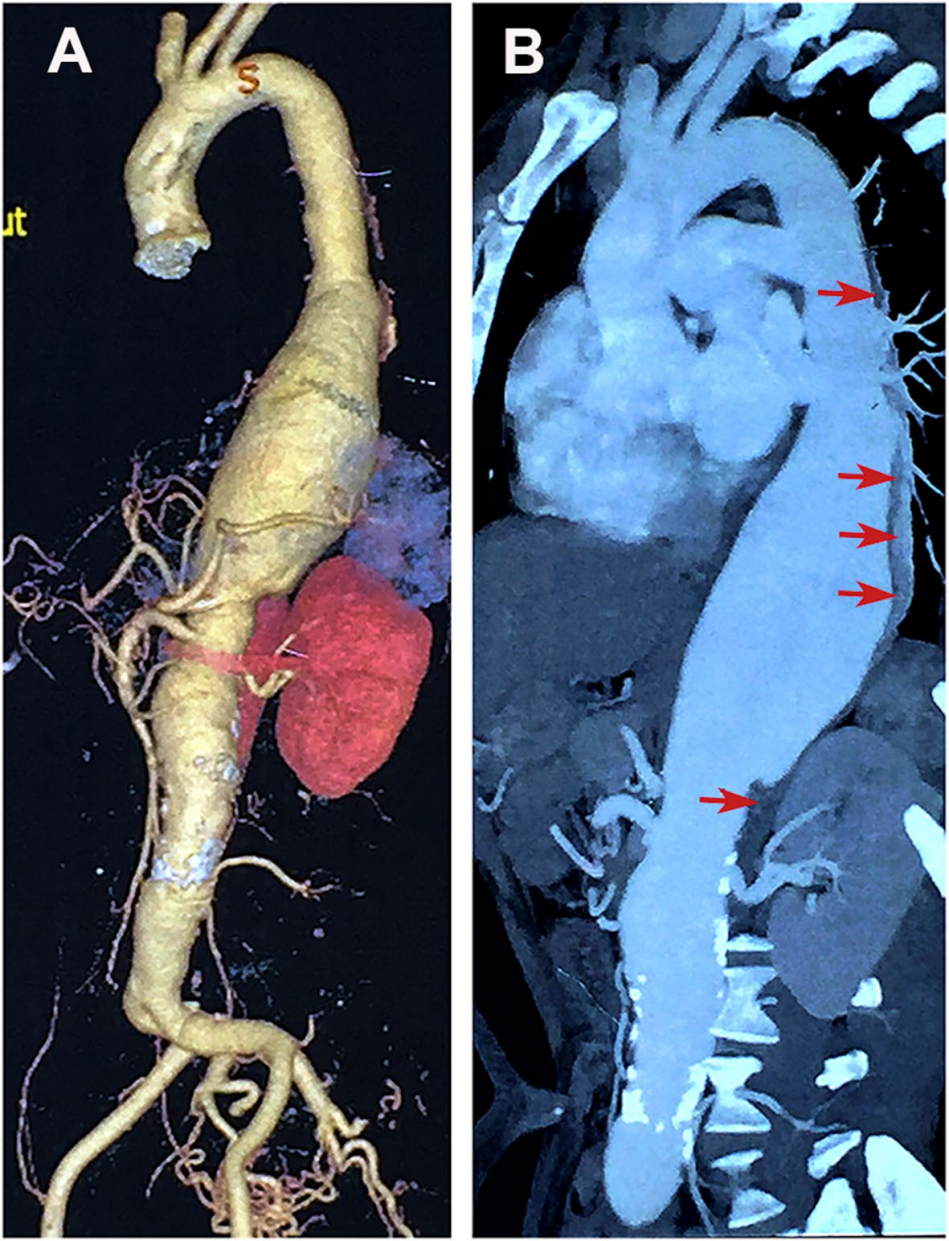
Preoperative reconstructed computed tomography images of the patient (**A**) CTA with 3D reconstructions revealing a type III thoracoabdominal aortic aneurysm (TAAA). **B** 2D sagittal view of the extent III TAAA with aortic intramural hematoma (red arrow)

**Fig. 2:**
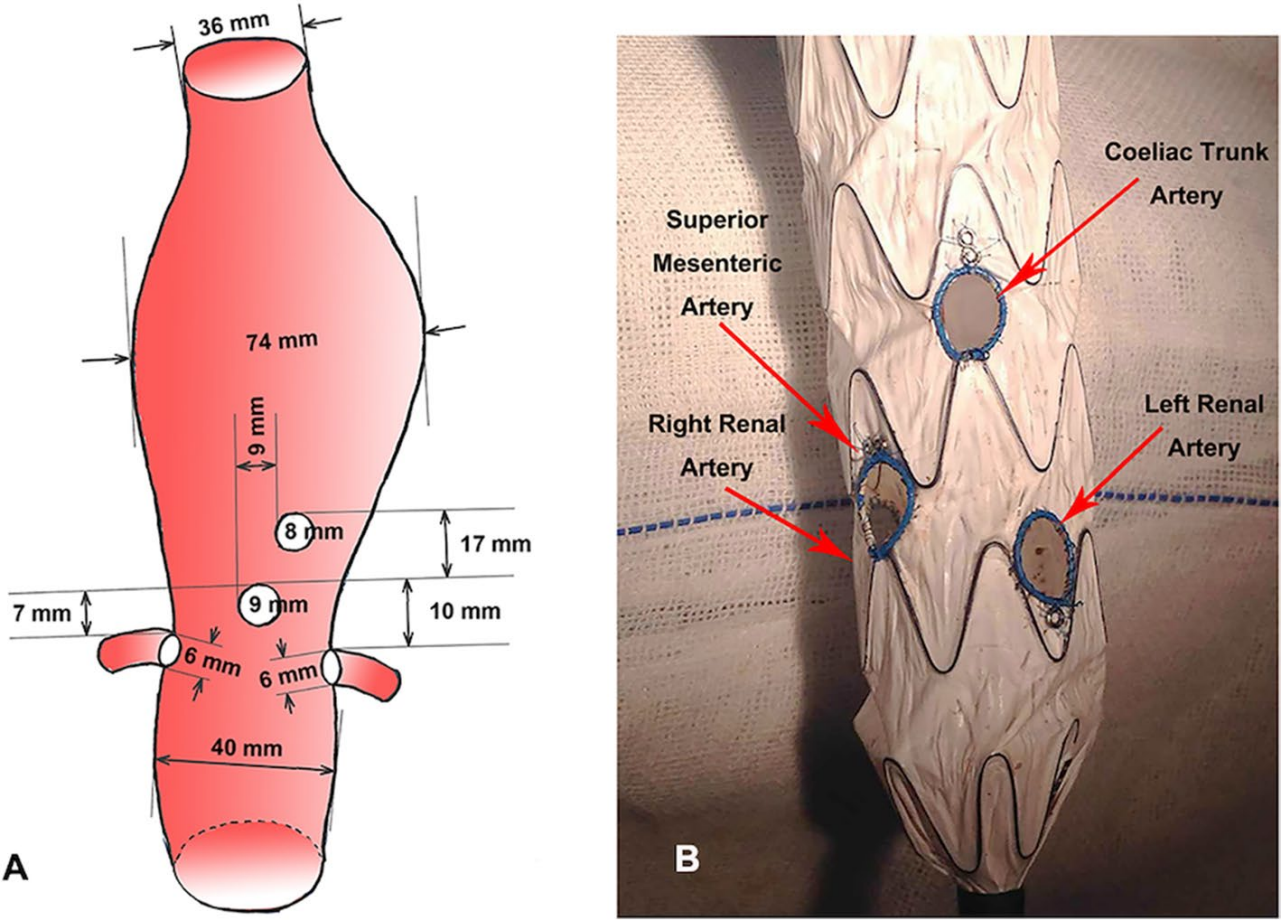
Modification of the Ankura II 38*200 tapering endograft. **A** Illustrations of data analysis based on CT measurement, the corresponding distance between four visceral arteries was demonstrated, using the superior mesenteric artery (SMA) ostium as reference. **B** Surgeon-modified fenestrated stent graft with four reinforced fenestrations to revascularize the visceral vessels

A 55-year-old female was urgently admitted to our hospital for acute backache and abdominal pain for 48 h. Following the completion of an emergent computed tomography angiography (CTA), an extent III TAAA with aortic intramural hematoma (Fig. 1A, B) was found, with a maximum diameter of 36 mm in the proximal descending aorta, a maximum diameter of 74 mm at the distal descending aorta level, 40 mm at the level of visceral artery and 30 mm from inferior mesenteric artery level to 3 cm distal to the bifurcation of abdominal aorta (Fig. 2A).

The patient had a history of hypertension, diabetes, chronic renal insufficiency and coronary disease with cardiac insufficiency. In addition, the patient had taken clopidogrel and aspirin for acute chest pain before admission. Given her multiple comorbidities, she was therefore considered at high surgical risk and unfit for open repair. After extensive discussion with the patient and her family, they wished to proceed with a repair that offered the lowest possible risk and minimally-invasive approach. Therefore, the thoracoscope-assisted fixation and SMFEG was finally chosen under informed consent for comprehensive treatment.

### Thoracoscope-assisted fixation

Surgery was performed with general anesthesia in the endovascular operating room. A 3-port technique was performed through a left video-assisted thoracoscopic approach. One 10-mm trocar was placed in the sixth intercostal space in mid-axillary line to accommodate thoracoscopic camera. And two 15-mm ports for operating were placed into the fourth intercostal space.

**Fig. 3:**
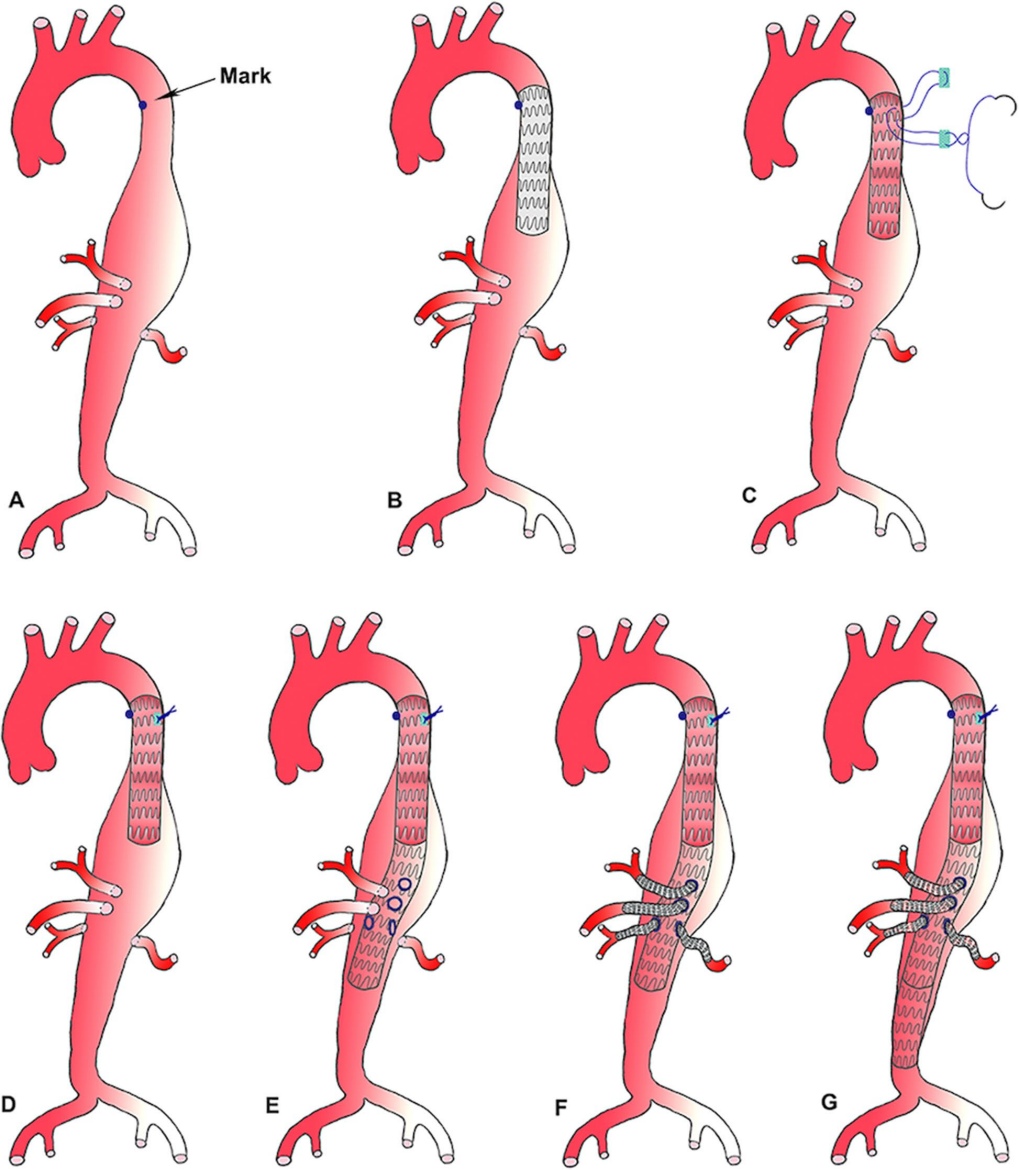
Schematic of steps of the thoracoscope-assisted fixation technique and implantation of the Surgeon–modified fenestrated stent graft (PMSG) with 4 branches reconstruction. **A** A metal mark was fixed on the adventitia of the proximal descending aorta. **B** A Ankura II endograft was deployed proximally. **C** A double needle suture with 2 felt pledgets was placed. **D** The suture was knotted and the fixation of the endograft was finished. **E** The PMSG was deployed with perfect apposition between the fenestrations and the target visceral artery. **F** Each visceral artery was sequentially stented using Viabahn self-expandable stent. **G** An Ankura cuff stent was deployed to seal the distal landing zone

Then a metal mark was fixed on the adventitia of the proximal descending aorta (Fig. 3A). Both femoral arteries were retrogradely punctured. Heparin was given intravenously, as an Ankura II tampering stent-graft 38*160 (Lifetech Scientific, China) was introduced over an Lunderquist super-stiff guidewire (Cook Inc, Bloomington, Ind). The 160-mm long main body of the Ankura II stent-graft is tapered; the diameter is 38 mm at the top and 34 mm at the bottom. Using the optimal C-arm position, the delivery system was advanced to orient the top markers of the stent-graft just over the level of the metal mark on the adventitia, then the first stent-graft was fully deployed (Fig. 3B). After insertion of the thoracoscope again, a double needle suture (4–0 Prolene) with 2 felt pledgets was placed on horizontal mattress type. Under radiation, the double needle was penetrated both the aortic wall and stent-graft and was knotted tightly (Fig. 3C-D).

### Device modification

The second Ankura II 38*200 tapering endograft, 200 mm long main body with 38 mm diameter at the top and 30 mm at the bottom, was partially deployed on a back table and four modified fenestrations was positioned according to the measurements of the CT scan analysis. To calculate the position of fenestrations, the superior mesenteric artery (SMA) ostium was considered the primary reference point (Fig. 2A). The four fenestrations to incorporate the celiac trunk, superior mesenteric artery (SMA), left renal artery (LRA) and right renal artery (RRA) graft were normally constructed in proper size. Celiac and SMA fenestrations are 9 mm in diameter located in the 01:00 and 12:00 clock positions, and RRA and LRA are 6 mm located at 9:00 and 03:00 positions, respectively. Each fenestration was reinforced by a radiopaque wire and marker using 6–0 prolene stitches, to prevent tearing or fraying of the graft fabric and to allow intraoperative orientation under fluoroscopy (Fig. 2B).

### Device orientation and deployment

**Fig. 4:**
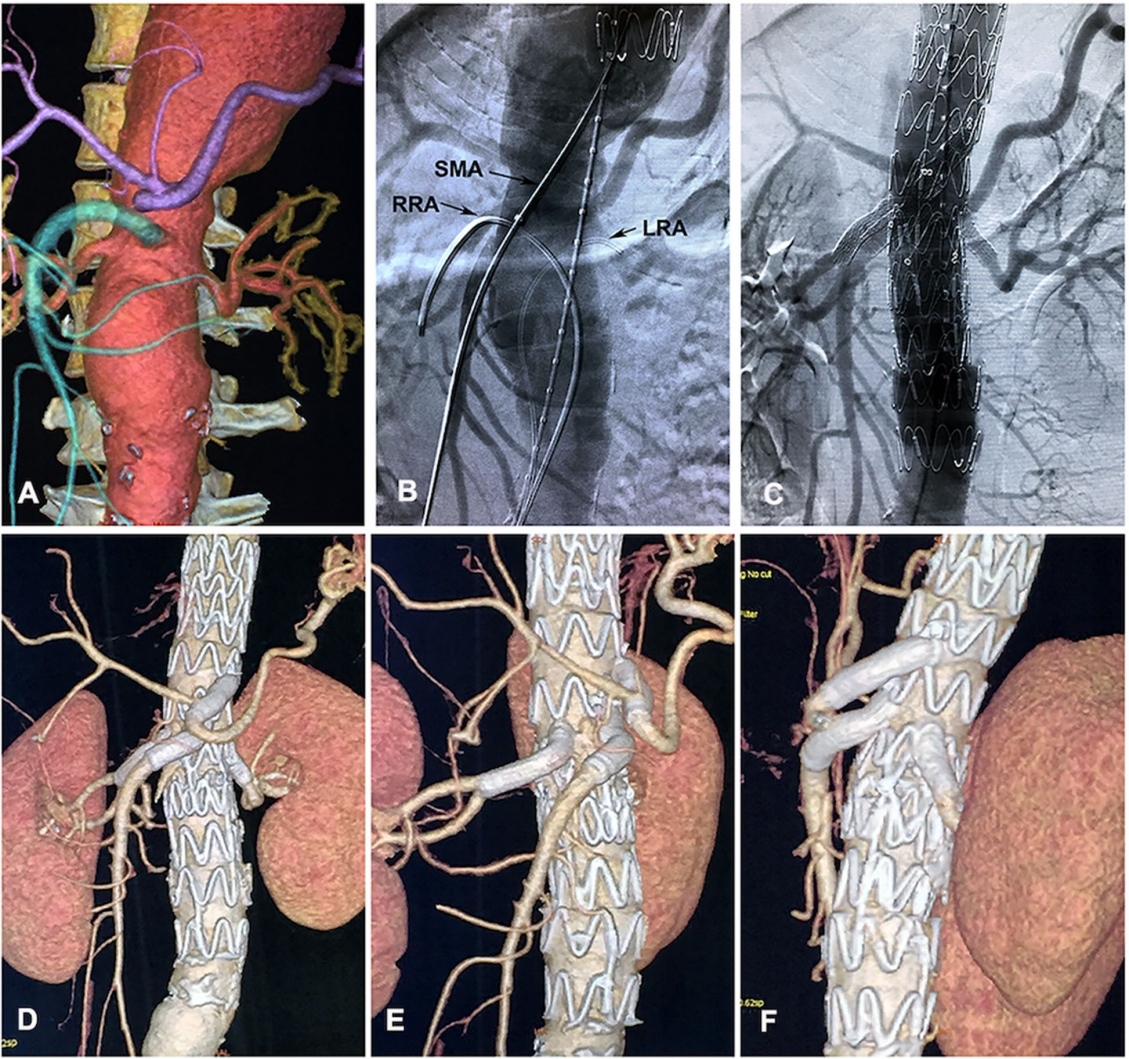
Pre-, intra-, and postoperative image of implantation of the Surgeon- modified fenestrated stent graft (PMSG) with 4 branches reconstruction. **A** Preoperative CTA of four visceral arteries. **B** Intraoperative angiography demonstrates catheterization of three visceral arteries. **C** Intraoperative angiography demonstrates implantation of PMSG with 4 branches reconstruction. **D**, **E**, **F** Postoperative CTA of 4 branches reconstruction in sagittal, right anterior oblique and left anterior oblique position, respectively

Using 5F catheter coude (Cordis Corp, Bridgewater, NJ), we catheterized the SMA, LRA and RRA through the access from left brachial and left femoral artery (Fig. 4B). Precatheterization of the SMA, LRA and RRA were used to be reference for the orientation and deployment of the endograft. Then the fenestrated endograft was reconstrained and introduced through the right femoral access. When advancing at the level of visceral artery, the endograft was oriented extracorporeally by rotating the SMA, LRA and RRA fenestration radiopaque markers and deployed with perfect apposition between the fenestrations and the target visceral artery (Fig. 3E).

### Target vessel stenting

The selective catheters were sequentially withdrawn from branch arteries and used to regain access to the fenestrations and target vessel. Hydrophilic sheaths (8 Fr) were advanced into the target vessels over stiff 0.035-inch guidewires. Each vessel was sequentially stented using Viabahn self-expandable stent (VSXS, W. L. Gore & Associates, Flagstaff, AZ, USA). A 9*59 mm VSXS was deployed in the celiac trunk artery and then post-dilated with a 10*30 mm balloon in its distal portion and the fenestrated ostium. Then a 10*59 mm VSXS for SMA and two 6*59 mm VSXSs for both renal arteries were deployed and post-dilated using the same method, respectively (Figs. 3F and 4). An Ankura cuff stent 32*80 was deployed in the distal abdominal aortic artery (Fig. 3G).

The attachment sites between the main body of the Ankura stent-graft, and the proximal and distal landing zones were dilated using Coda balloon (Cook Medical), followed by completion angiography, which confirmed widely four branch fenestrated stent graft and distal abdominal aortic artery with no endoleak (Fig. 4C).

**Fig. 5:**
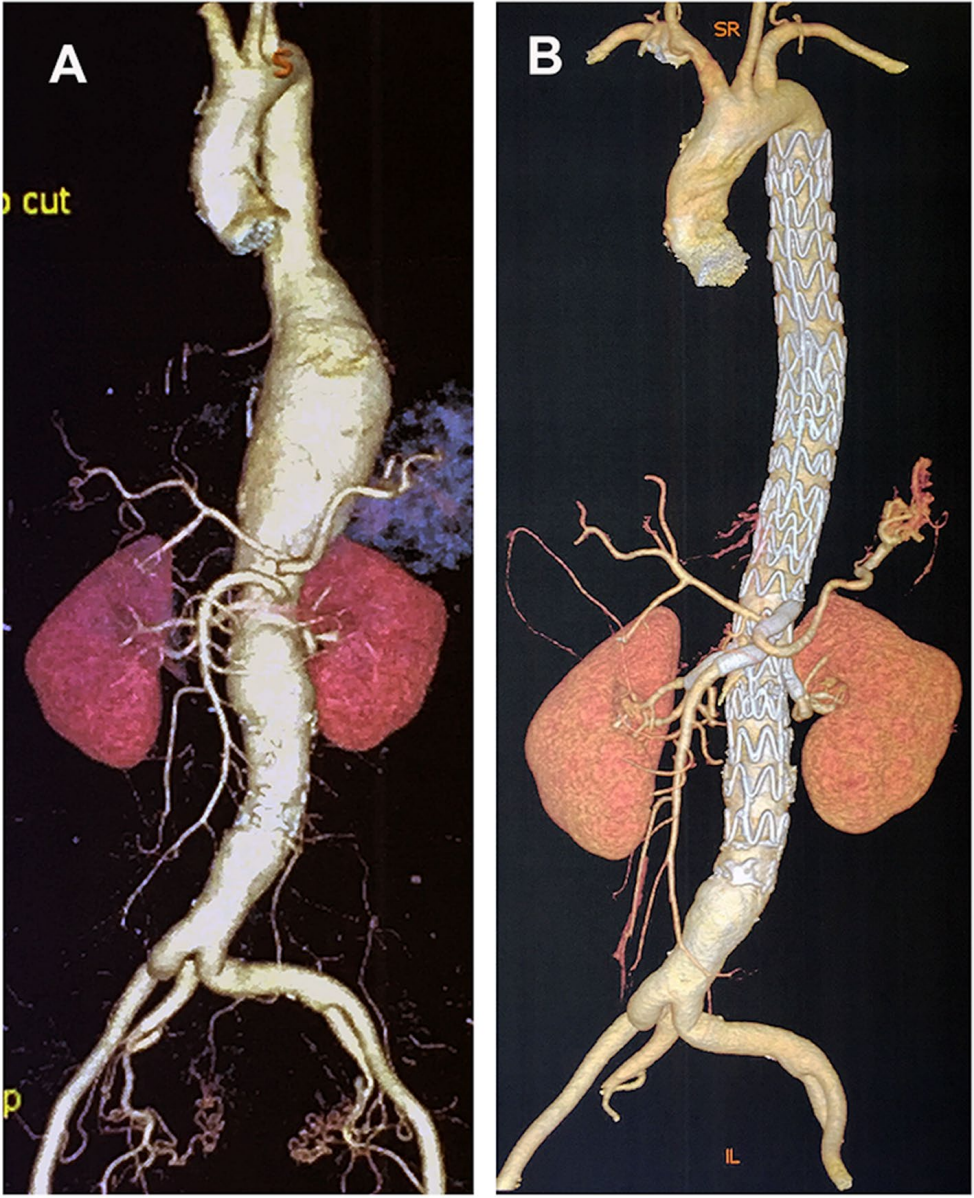
Pre- and postoperative image of the extent III thoracoabdominal aortic aneurysm (TAAA). **A** Coronal view of the preoperative extent III TAAA. **B** Postoperative CTA examination in one-month follow-up confirmed patency of all four branches with no endoleak

The total operative time was 6 h, fluoroscopy time was 56 min, and 88 mL of iodinated contrast was administered. The patient had an uncomplicated postoperative course and was discharged on the second postoperative week. CTA obtained at one week and at six months postoperatively revealed no endoleak and widely patent visceral branches (Fig. 5B).

## Discussion and Conclusion

The current data suggest that FEVAR is an effective treatment with high technique success for selected patients at high risk of open repair of extent TAAA [3]. However, high device costs and long manufacturing delays limit its applicability. And the “off-the-shelf” fenestrated/branched endografts are not yet widely available in many countries. Thus, SMFEG is an alternative for branch vessel preservation of symptomatic TAAA [4].

In these circumstances, we have to use SMFEG by modifying commercially available aortic endovascular stent-graft with reinforced fenestrations. In our opinion, SMFEG with branches reconstruction for the visceral vessels is a viable therapeutic alternative for high-risk patients unfit for open surgery. However, total TEVAR may be hampered by late dilatation of the proximal landing zone, which can lead to endograft migration and potentially catastrophic consequences. In particular, the proximal descending aortic pathology of the patient described in this report, characterized by an inverted conical and coarsened shape with a maximum diameter of 36 mm in the proximal landing zone with intramural hematoma, indicated that she was not an ideal candidate for total endovascular technique with SMFEG.

For this reason, a new hybrid approach combining thoracoscope-assisted fixation with SMFEG was decided. The first endograft was fixed in the proximal descending aorta by a suture through video-assisted thoracoscopy, avoiding endografts migration even if the aorta at proximal landing zone gradually dilated in the future. To the best of our knowledge, our institution is the first to describe the combination of thoracoscope-assisted fixation technique and FEVAR in treatment of extent TAAA with aortic intramural hematoma.

The main consideration for using thoracoscopic fixation was that if we didn't fix the first stent graft in advance, we had to worry about the possibility of the second stent graft affecting the first stent graft during release. That is, after the first stent graft was fully released, when we were implanting the fenestrated stent graft, if there was an inaccurate alignment between the fenestrations and the target visceral artery, we would have to repeatedly pull the fenestrated stent graft to realign it, and if we hadn't fixed the first stent in advance, because the proximal segment of the fenestrated stent graft is attached to the first stent graft, combined with the continuous impact of arterial blood flow, the first stent graft may passively follow the fenestrated stent graft and shift or even fall down into the aneurysm.

The advantages of thoracoscope-assisted fixation may include, 1) without increasing the surgical trauma, because only 3 ports (10–15 mm mini-incision) were better placed in the intercostal space of left chest to complete the sutures with video assisted thoracoscopy; 2) avoiding endograft migration even if the aorta at proximal landing zone gradually dilated in the future, so as to avoiding potentially catastrophic consequences. However, fixation of the stent graft is unlikely to prevent landing zone dilatation and endoleak. Some experts have suggested using a "band" around the aorta to prevent endograft migration and endoleak. However, we were concerned that thoracoscopic fixation would be easier than thoracoscopic "banding", which involves more complex anatomical procedures.

In addition, modular separation, following component migration in the overlapping zone of TEVAR, is recognized as a potential source of late failure [5]. However, the main bodies of current endografts used for this patient are tapering design, which may have the potential to reduce endograft migration and thereby protect branch vessels.

In conclusion, the technique described here, combining thoracoscopic fixation and SMFEG with branch reconstruction, represents an interesting and viable alternative for selected high-risk patients with extent III TAAA and aortic intramural haematoma. Longer follow-up is required to determine the success of this approach.

## Data Availability

The data is available and can be sought with the authors on request.

## Abbreviations

TAAA: Thoracoabdominal aortic aneurysm
FEVAR: Fenestrated endovascular aortic repair
EVAR: Endovascular aortic repair
SMFEG: Surgeon-modified fenestrated endovascular graft
CTA: Computed tomography angiography
